# Supplementary material for: Mindfully and confidently digital: A mixed methods study on personal resources to mitigate the dark side of digital working
Source: PLoS One. 2024 Feb 23;19(2):e0295631. doi: 10.1371/journal.pone.0295631 (PMC10889626; doi:10.1371/journal.pone.0295631)
Supplement: S1 Appendix — (DOCX) [file pone.0295631.s001.docx]

**S1 Appendix**

# S1 Appendix. Construct definitions and key references

| **Construct** | **Definition** | **Key references** |
| --- | --- | --- |
| Digital workplace stress | A negative psychological state experienced by employees as a result of perceived digital workplace stressors such as overload, interruptions, invasion, and the pressure and pace of digital work; and a lack of resources to cope with these demands. | [1-5] |
| Digital workplace overload | Employee perceptions of excessive and burdensome levels of technology and technology features as well as information and communication received in the digital workplace, resulting in elevated stress and burnout, and lower productivity. | [6-7] |
| Digital workplace anxiety | A sense of tension, uneasiness, apprehension and even aversion that may be felt by employees in response to the necessity to use digital workplace technologies. | [8-12] |
| Digital workplace Fear of Missing Out (FoMO) | Apprehension experienced by employees when working in the digital workplace that they may be disadvantaged in some way in their work or career due to missing out on important informational or relational opportunities. | [13-14] |
| Digital workplace addiction | Excessive and compulsive use of digital workplace technology that results in negative well-being outcomes for workers. | [8, 15] |
| Trait mindfulness (TM) | A secondary personality trait which promotes a state of consciousness involving paying attention to and being aware of both internal and external phenomena occurring in the present moment and doing so in a non-judgmental manner. | [16-19] |
| Digital workplace confidence (DWC) | Results from individuals’ perceptions of self-efficacy in terms of how they apply existing digital skills to using novel digital workplace tools or features and in an independent manner. | [20-22] |
| Burnout | A negative employee well-being indicator comprising exhaustion due to intense strain at work and disengagement from work itself as reflected in a negative attitude towards it. | [23-24] |
| Health | One’s overall state of health (a state of sickness versus one of health) including mental health (feeling nervous and depressed versus peaceful, happy and calm). | [25] |

**S1 Appendix References**

1. Brod C. Technostress: The Human Cost of the Computer Revolution. Addison-Wesley Publishing Company, Reading, USA; 1984.
2. Ragu-Nathan TS, Tarafdar M, Ragu-Nathan BS, Tu Q. The consequences of technostress for end users in organizations: Conceptual development and empirical validation. Information Systems Research. 2008;19(4):417-433. doi:10.1287/isre.1070.0165.
3. Salanova M, Llorens S, Cifre E, Martínez IM, Schaufeli WB. Perceived collective efficacy, subjective well-being and task performance among electronic work groups: An experimental study. Small Group Research. 2003;34(1):43-73. doi:10.1177/1046496402239577.
4. Arnetz BB, Wiholm C. Technological stress: Psychophysiological symptoms in modern offices. J Psychosom Res. 1997;43(1):35-42. <https://doi.org/10.1016/S0022-3999(97)00083-4>
5. Galluch PS, Grover V, Thatcher JB. Interrupting the workplace: Examining stressors in an information technology context. J Assoc Inf Syst. 2015;16(1):1-47.
6. Karr-Wisniewski P, Lu Y. When more is too much: Operationalizing technology overload and exploring its impact on knowledge worker productivity. Comput Hum Behav. 2010;26(5):1061-1072. <http://doi.org/10.1016/j.chb.2010.03.008>
7. Karr P, Lu Y. Information technology and knowledge worker productivity: a taxonomy of technology crowding. Americas Conference on Information Systems 2007 Proceedings. Colorado, United States.
8. Salanova M, Llorens S, Cifre E. The dark side of technologies: Technostress among users of information and communication technologies. International Journal of Psychology. 2013;48(3):422-436. doi:10.1080/00207594.2012.680460.
9. Fernández-Batanero JM, Román-Graván P, Reyes-Rebollo MM, Montenegro-Rueda M. Impact of educational technology on teacher stress and anxiety: A literature review. Int J Environ Res Public Health. 2021;18(2):548. <http://doi.org/10.3390/ijerph18020548>
10. Beckers JJ, Schmidt HG. The structure of computer anxiety: A six-factor model. Comput Human Behav. 2001;17(1):35-49. <https://doi.org/10.1016/S0747-5632(00)00036-4>
11. Leso T, Peck KL. Computer anxiety and different types of computer courses. J Educ Comput Res. 1992;8(4):469-478. <https://doi.org/10.2190/Q1TJ-8JCU-LDAP-84H8>
12. Powell AL. Computer anxiety: Comparison of research from the 1990s and 2000s. Computers in Human Behavior. 2013;29(6):2337-2381. doi:10.1016/j.chb.2013.05.012.
13. Budnick CJ, Rogers AP, Barber LK. The fear of missing out at work: examining costs and benefits to employee health and motivation. Comput Human Behav. 2020;104:1-13. <https://doi.org/10.1016/j.chb.2019.106161>
14. Fridchay J, Reizer A. Fear of Missing out (FOMO): Implications for Employees and Job Performance. J Psychol. 2022;156(4):257-277. <http://doi.org/10.1080/00223980.2022.2034727>
15. Del Líbano M, Llorens S, Salanova M, Schaufeli WB. Validity of a brief workaholism scale. Psicothema. 2010;22(1):143-150.
16. Brown KW, Ryan RM. The benefits of being present: mindfulness and its role in psychological well-being. J Pers Soc Psychol. 2003;84(4):822. <http://doi.org/10.1037/0022-3514.84.4.822>
17. Kabat-Zinn J. Wherever you go there you are: Mindfulness meditation in everyday life. New York, NY: Hyperion; 1994.
18. Shapiro SL, Carlson LE, Astin JA, Freedman B. Mechanisms of mindfulness. Journal of Clinical Psychology. 2006;62(3):373-386. doi:10.1002/jclp.20237.
19. Dane E, Brummel BJ. Examining workplace mindfulness and its relations to job performance and turnover intention. Hum Relat. 2014;67(1):105-128. <http://doi.org/10.1177/0018726713487753>.
20. Compeau DR, Higgins CA. Computer self-efficacy: Development of a measure and initial test. MIS Q. 1995;19(2):189-211. <http://doi.org/10.2307/249688>
21. Maier C, Wirth J, Laumer S, Weitzel T. Personality and Technostress: Theorizing the influence of IT mindfulness [Conference paper]. Proceedings of the 38th International Conference on Information Systems (ICIS), Seoul, South Korea; 2017.
22. Rasool T, Warraich NF, Sajid M. Examining the Impact of Technology Overload at the Workplace: A Systematic Review. SAGE Open. 2022;12(3):21582440221114320. doi:10.1177/21582440221114320.
23. Demerouti E, Bakker AB, Vardakou I, Kantas A. The convergent validity of two burnout instruments: A multitrait-multimethod analysis. Eur J Psychol Assess. 2003;19(1):12. <http://doi.org/10.1027/1015-5759.19.1.12>
24. Schaufeli WB, Bakker AB. Job demands, job resources, and their relationship with burnout and engagement: A multi‐sample study. Journal of Organizational Behavior. 2004;25(3):293-315. doi:10.1002/job.248.
25. Ware Jr JE, Sherbourne CD. The MOS 36-item short-form health survey (SF-36): I. Conceptual framework and item selection. Medical care. 1992:473-483.
