# Supplementary material for: Mindfully and confidently digital: A mixed methods study on personal resources to mitigate the dark side of digital working
Source: PLoS One. 2024 Feb 23;19(2):e0295631. doi: 10.1371/journal.pone.0295631 (PMC10889626; doi:10.1371/journal.pone.0295631)
Supplement: S2 Appendix — (DOCX) [file pone.0295631.s002.docx]

**S2 Appendix**

# S2 Appendix. Measurement items

*Digital workplace stress [1]*

1. I feel drained from activities that require me to use digital workplace technologies.
2. I feel tired from my digital workplace activities.
3. Working all day with digital workplace technologies is a strain for me.
4. I feel burned out from my digital workplace activities.

*Digital workplace overload [2]*

1. I am often distracted by the excessive amount of information available to me for business decision making.
2. I find that I am overwhelmed by the amount of information I have to process on a daily basis.
3. Usually, my problem is with too much information to synthesize instead of not having enough information to make decisions.
4. I am often distracted by features that are included in applications I use for my job but are not necessary to perform my job duties.
5. I am often less productive because of poor user interface design in digital workplace applications I use to support my daily business activities.
6. I find that most digital workplace applications I use at work handle too many tasks poorly instead of too few tasks very well.
7. Many digital workplace applications I use at work tend to try to be too helpful which makes performing my job even harder.
8. The digital workplace applications I use for work are often more complex than the tasks I have to complete using these packages.
9. I feel that in a less connected environment, my attention would be less divided allowing me to be more productive.
10. I often find myself overwhelmed because technology has allowed too many other people to have access to my time.
11. I waste a lot of my time responding to emails and messages in collaboration environments that are business-related but not directly related to what I need to get done.

The availability of electronic communication has created more of an interruption than it has improved communications.

*Digital workplace anxiety [1]*

1. I feel apprehensive about using digital workplace applications.
2. It scares me to think that I could lose a lot of information using digital workplace applications by hitting the wrong key.
3. I hesitate to use digital workplace applications for fear of making mistakes I cannot correct.
4. Digital workplace applications are somewhat intimidating to me.

*Fear of missing out in the digital workplace [3]*

When working in the digital workplace…

1. …I worry that I might miss important work-related updates.
2. …I worry that I might miss out on valuable work-related information.
3. …I worry that I will miss out on important work-related news.
4. …I worry that I will miss out on important information that is relevant to my job.
5. …I worry that I will not know what is happening at work.
6. …I get anxious that I will miss out on an opportunity to make important business connections.
7. …I am constantly thinking that I might miss opportunities to strengthen business contacts.
8. …I am constantly thinking that I might miss opportunities to make new business contacts.
9. …I worry that I will miss out on networking opportunities that my co-workers will have.
10. …I fear that my co-workers might make business contacts that I won’t make.

*Digital workplace addiction [4]*

1. I feel I use digital workplace technologies in excess in my life.
2. I seem to have an inner compulsion to use digital workplace technologies in whatever place and time.
3. I find myself staying connected to work using the digital workplace after my co-workers have called it quits.
4. I feel guilty when I’m not available to colleagues using the digital workplace when I take time off work.

Note: items 3 and 4 are new items added for the present study and based on Del Libano et al. (2010**)** which is also the basis for the above authors’ original 2 items.

*Trait mindfulness [5, 6]*

1. I could be experiencing some emotion and not be conscious of it until sometime later.
2. I break or spill things because of carelessness, not paying attention, or thinking of something else.
3. I find it difficult to stay focused on what’s happening in the present.
4. I tend to walk quickly to get where I’m going without paying attention to what I experience along the way.
5. I tend not to notice feelings of physical tension or discomfort until they really grab my attention.
6. It seems I am “running on automatic,” without much awareness of what I’m doing.
7. I rush through activities without being really attentive to them.
8. I get so focused on the goal I want to achieve that I lose touch with what I’m doing right now to get there.
9. I do jobs or tasks automatically, without being aware of what I'm doing.
10. I drive or walk places on ‘automatic pilot’ and then wonder why I went there.
11. I find myself doing things without paying attention.

*Digital workplace confidence [7]*

I could complete the task or job using digital workplace technologies...

1. ...if there was no one around to tell me what to do
2. ...if I had never used an application like it before.
3. ... if I had only the application manual for reference.
4. ...if I had seen someone else using it before trying it myself.
5. ...if I could call someone for help if I got stuck.
6. ...if someone else had helped me get started.
7. ...if I had a lot of time to complete the job for which the application was provided.
8. ...if I had a built-in help facility for assistance.
9. ...if someone showed me how to do it first.
10. …if I had used similar applications before this one to do the same job.

*Burnout [8]*

1. I always find new and interesting aspects in my work.
2. There are days when I feel tired before I arrive at work.
3. It happens more and more often that I talk about my work in a negative way.
4. After work, I tend to need more time than in the past in order to relax and feel better.
5. I can tolerate the pressure of my work very well.
6. Lately, I tend to think less at work and do my job almost mechanically.
7. I find my work to be a positive challenge.
8. During my work, I often feel emotionally drained.
9. Over time, one can become disconnected from this type of work.
10. After working, I have enough energy for my leisure activities.
11. Sometimes I feel sickened by my work tasks.
12. After work, I usually feel worn out and weary.
13. This is the only type of work that I can imagine myself doing.
14. Usually, I can manage the amount of my work well.
15. I feel more and more engaged with my work.
16. When I work, I usually feel energised.

*Health [9]*

1. In general, would you say your health is: Excellent, Very good, Good, Fair, Poor.

How TRUE or FALSE is each of the following statements for you?

1. I seem to get sick a little easier than other people.
2. I am as healthy as anybody I know.
3. I expect my health to get worse.
4. My health is excellent.

These questions are about how you feel and how things have been with you during the past 4 weeks. For each question, please give the one answer that comes closest to the way you have been feeling. How much of the time during the past 4 weeks –

1. have you been a very nervous person?
2. have you felt so down in the dumps that nothing could cheer you up?
3. have you felt calm and peaceful?
4. have you felt downhearted and blue?
5. have you been a happy person?

**S2 Appendix References**

1. Venkatesh V, Morris MG, Davis GB, Davis FD. User acceptance of information technology: Toward a unified view. MIS quarterly. 2003:425-478. doi:10.2307/30036540.
2. Karr-Wisniewski P, Lu Y. When more is too much: Operationalizing technology overload and exploring its impact on knowledge worker productivity. Comput Hum Behav. 2010;26(5):1061-1072. <http://doi.org/10.1016/j.chb.2010.03.008>
3. Budnick CJ, Rogers AP, Barber LK. The fear of missing out at work: examining costs and benefits to employee health and motivation. Comput Human Behav. 2020;104:1-13. <https://doi.org/10.1016/j.chb.2019.106161>
4. Salanova M, Llorens S, Cifre E. The dark side of technologies: Technostress among users of information and communication technologies. International Journal of Psychology. 2013;48(3):422-436. doi:10.1080/00207594.2012.680460.
5. Brown KW, Ryan RM. The benefits of being present: mindfulness and its role in psychological well-being. J Pers Soc Psychol. 2003;84(4):822. <http://doi.org/10.1037/0022-3514.84.4.822>
6. Chiesi F, Donati MA, Panno A, Giacomantonio M, Primi C. What about the different shortened versions of the Mindful Attention Awareness Scale? Psychol Rep. 2017;120(5):966-990. <http://doi.org/10.1177/0033294117711132>.
7. Compeau DR, Higgins CA. Computer self-efficacy: Development of a measure and initial test. MIS Q. 1995;19(2):189-211. <http://doi.org/10.2307/249688>
8. Demerouti E, Bakker AB, Vardakou I, Kantas A. The convergent validity of two burnout instruments: A multitrait-multimethod analysis. Eur J Psychol Assess. 2003;19(1):12. <http://doi.org/10.1027/1015-5759.19.1.12>
9. Ware Jr JE, Sherbourne CD. The MOS 36-item short-form health survey (SF-36): I. Conceptual framework and item selection. Medical care. 1992:473-483.
