# Supplementary material for: Mindfully and confidently digital: A mixed methods study on personal resources to mitigate the dark side of digital working
Source: PLoS One. 2024 Feb 23;19(2):e0295631. doi: 10.1371/journal.pone.0295631 (PMC10889626; doi:10.1371/journal.pone.0295631)
Supplement: S3 Appendix — (DOCX) [file pone.0295631.s003.docx]

**S3 Appendix**

# S3 Appendix. Figures for mediation analyses





# S3 Figure 1. Model of digital workplace stress as a predictor of health, mediated by burnout

*Note.* ***** *p* <.05, ** *p* < .01. *B* = standardized beta coefficients. *N* = 140, with pairwise deletion for missing data.





**S3 Figure 2.** Model of digital workplace overload as a predictor of health, mediated by burnout

*Note.* ***** *p* <.05, ** *p* < .01. *B* = standardized beta coefficients. *N* = 140, with pairwise deletion for missing data.




**S3 Figure 3.** Model of digital workplace anxiety as a predictor of health, mediated by burnout

*Note.* ***** *p* <.05, ** *p* < .01. *B* = standardized beta coefficients. *N* = 140, with pairwise deletion for missing data.





**S3 Figure 4.** Model of digital workplace FoMO as a predictor of health, mediated by burnout

*Note.* ***** *p* <.05, ** *p* < .01. *B* = standardized beta coefficients. *N* = 140, with pairwise deletion for missing data.
